# Chronic Stress Indicator: A Novel Tool for Comprehensive Stress Analysis

**DOI:** 10.3390/ijerph21030302

**Published:** 2024-03-05

**Authors:** Matthew Hill, Sayed Mostafa, Emmanuel Obeng-Gyasi

**Affiliations:** 1 Department of Built Environment, North Carolina A&T State University, Greensboro, NC 27411, USA; 2 Environmental Health and Disease Laboratory, North Carolina A&T State University, Greensboro, NC 27411, USA; 3 Department of Mathematics and Statistics, North Carolina A&T State University, Greensboro, NC 27411, USA

**Keywords:** stress, allostatic load, allostasis, cumulative

## Abstract

Extensive research has highlighted the strong association between chronic stress and negative health outcomes. This relationship is influenced by various factors, including sociobehavioral, environmental, and genetic and epigenomic forces. To comprehensively assess an individual’s stress levels, we propose the development of the Chronic Stress Indicator (CSI), a novel comprehensive multifaceted tool that incorporates key biological, anthropometric, behavioral, and socioeconomic factors. The objective of this study is to assess the effectiveness of the CSI compared to Allostatic Load (AL), a type of chronic stress, in identifying health issues related to stress. The objective of this research is to evaluate the performance of the Chronic Stress Indicator (CSI) versus Allostatic Load (AL) in detecting adverse health outcomes within the U.S. demographic aged 20–49. The information used for this study was sourced from the National Health and Nutrition Examination Survey (NHANES), carried out from 2001 to 2004. Logistic regression modeling was employed to calculate odds ratios and confidence intervals. The Wilcoxon rank-sum test was employed to assess differences in means, whereas the chi-square test, accompanied by Cramer’s V statistic, was used to examine the association among categorical variables. Additionally, the relationship between continuous variables was analyzed using Pearson’s correlation coefficient. Our association tests show that the length of occupation activity and health status were among the strongest associations to CSI risk. Based on our logistic regression models, age and sex were found to be significant factors in determining AL. We also found that age, smoking, and longest occupation activity were significant factors of CSI risk. These findings suggest a need for individuals to limit smoking as it may lead to higher overall stress despite its common use as a coping mechanism for stress. We should also review the level of occupational activity a job has before continuously working on it as this may also lead to higher cumulative stress.

## 1. Introduction

Chronic stress is recognized for its comprehensive impact on various aspects of physical and mental health, including cardiovascular well-being, immune system function, cognitive performance, sleep quality, and overall quality of life [1,2]. Indeed, the multifaceted impact of stress is such that it can affect mood and have profound consequences on various physiological systems within the human body. One key mechanism through which chronic stress exerts its influence is the activation of the hypothalamic–pituitary–adrenal (HPA) axis, a complex neuroendocrine pathway involved in the stress response [3].

The HPA axis plays a critical role in regulating the body’s response to stressors, including the release of stress hormones such as cortisol. Prolonged activation of the HPA axis due to chronic stress can disrupt the delicate balance of cortisol production, leading to dysregulation of the stress response system [3]. This dysregulation has been associated with poor health outcomes.

Previous studies have established Allostatic Load (AL) as an indicator of this chronic stress [2,4,5,6]. Allostatic load is a concept that describes the cumulative “wear and tear” on the body as a result of exposure to repeated or chronic stress. Introduced by McEwen and Stellar in 1993, this idea revolves around the body’s physiological systems and their ability to adapt to stressors, albeit with a long-term cost to the body. At the core of allostatic load are biological pathways, as it emphasizes the role of biological markers from various systems, including the cardiovascular, metabolic, and immune systems. This notion of allostatic load is not just about the immediate response to stress but also about the accumulation of stress over time. While this has proved useful, it fails to include the impact of sociodemographic factors and behavioral choices when assessing chronic stress. 

There is a need to comprehensively produce an easy-to-use tool which captures the stress response. This is so because there is a need to capture individuals at high risk [7] for stress-related disease processes in order to be able to intervene and mitigate this risk. We use this principle to propose a tool for scoring excessive stress, the Chronic Stress Indicator (CSI), consisting of socioeconomic variables, biomarkers of health, and behavioral and lifestyle variables. Justification for the variables used in the construction of the CSI is based on the literature [7,8,9] with variables such as socioeconomic status [9], physical activity, and critical biomarkers from the cardiometabolic system used to capture the manifestation of the effects of prolonged stress.

Traditional operationalization of AL has focused on biomedical manifestations of stress. The development of a new stress analysis tool that integrates factors such as income, education, physical activity, alcohol, and tobacco use could lead to a more comprehensive and nuanced understanding of stress. This would consider the profound impact of socioeconomic factors on stress levels. For instance, lower income and educational attainment are frequently linked with higher stress [10,11,12], often due to financial instability, job insecurity, and limited access to necessary resources. By including income and education as key components, this would effectively recognize these critical socioeconomic determinants of health.

In addition to socioeconomic factors, this would also consider crucial lifestyle elements. For instance, engaging in physical activity significantly contributes to stress management [13,14]. Engaging in regular physical activity can help mitigate the adverse effects of stress on the body, while a sedentary lifestyle might worsen chronic stress. Thus, incorporating physical activity into the analysis would underscore the importance of lifestyle in stress management. Furthermore, this would also examine the use of alcohol and tobacco, which are commonly used as coping mechanisms for stress [15,16,17,18]. However, these substances can lead to increased health risks and contribute to worse health outcomes. By including alcohol and tobacco use, this could provide valuable insights into the complex relationship between substance use, stress, and overall health.

The purpose of this study is to then develop and assess the CSI and compare it to a traditional operationalization of chronic stress, such as Allostatic Load (AL). This study will demonstrate if integrating social and economic variables such as income and education, and behavioral variables such as physical activity along with cardiometabolic-related biomarkers better captures stress-related diseases. 

## 2. Materials and Methods

### 2.1. Population for the Study

The sample for this study came from the 2001–2004 NHANES cohort with individuals aged 20–49 years who had complete data for all indicators for both the AL and CSI indices included. The NHANES 2001–2004 employs a sophisticated, multi-stage, and stratified sampling method to collect data from non-institutionalized individuals in the U.S. For the purposes of this analysis, out of a total of 4161 participants sampled, 1063 were analyzed for AL and 921 for the CSI, contingent upon the availability of data.

### 2.2. Operationalization of Study Variables

In this research, demographic factors were collected with variables measured including age, gender, race/ethnicity, income level, levels of physical activity, educational background, and employment status. Other variables obtained using an in-home interview include cancer, chest pain, shortness of breath on stairs/incline, liver condition, and family history of diabetes, as well as hypertension/stroke. The study also included health-related variables collected using the mobile examination center as follows: cytomegalovirus (CMV) IgM, CMV IgG, Toxoplasma IgG, cancer antigen 125 (CA-125), Cancer antigen 15-3 (CA15-3), current health status, alcohol use, tobacco consumption, and the number of days physical as well as an indicator of mental health status. The laboratory procedures described in the NHANES Laboratory Procedure Manual [19] were performed to assess lab-based variables.

### 2.3. Overview of Research Variables and Associated Factors 

The study focuses on the analysis of primary outcome measures, including AL and the CSI. Investigated variables feature gender, annual family income, ethnicity, age, educational attainment, alcohol intake, smoking status, physical activity level, CMV IgG concentrations, SBP, DBP, TC levels, HDL levels, HBA1C, Albumin, triglycerides, BMI, creatinine clearance (CLCR), C-reactive protein (CRP), Toxoplasma IgG, symptoms such as chest pain and breathlessness on exertion, cancer presence, overall health condition, mental and physical health days, liver disorders, familial diabetes, and history of hypertension/stroke. It also examines current and longest-held job roles, activity levels at work (COA and LOA), along with markers like CA-125 and CA15-3. A categorization of occupations by activity level is provided in Appendix A for reference. In the categorization of occupations based on activity level, low occupational activity roles typically encompass professional, managerial, administrative, and clerical positions such as executives, managers, engineers, secretaries, and technicians. These roles are often characterized by sedentary tasks, intellectual or creative work, and decision-making responsibilities. In contrast, high occupational activity roles are primarily composed of manual labor and skilled trades, including construction workers, machine operators, and agricultural workers. These positions are physically demanding, involving significant on-your-feet tasks, manual skills, and outdoor labor. Additionally, high activity roles include service industry jobs like waiters and cooks, as well as protective service occupations, which require physical exertion and constant alertness [20,21,22]. Income was assessed as follows, ($0–$4999), ($5000–$9999), ($10,000–$14,999), ($15,000–$19,999), ($20,000–$24,999), ($25,000–$34,999), ($35,000–$44,999), ($45,000–$54,999), ($55,000–$64,999), ($65,000–$74,999), and (those earning more than $75,000). Participants who preferred not to choose a precise range for this variable were given two alternatives: “More than $20,000” and “Less than $20,000”. Subsequently, this variable was transformed into a binary indicator of poverty status using income data from the period 2001–2004. Education levels included less than 9th grade, 9–11th grade (including 12th grade with no diploma), High school graduate/General Educational Development (GED) or equivalent, some college or AA degree, and college graduate or above. Race/Ethnicity was classified into several groups: non-Hispanic White, non-Hispanic Black, Mexican American, Other Hispanic, and Other/Multiracial. Responses to questions about physical activity were recorded as either “yes” or “no” and “unable to do”. Current smoking status was recorded as “yes” for anyone who had smoked at least one cigarette in the previous month. Alcohol intake was marked as “yes” for individuals who had consumed 12 or more alcoholic beverages over the course of a year, and “no” for those who had not.

### 2.4. Operationalizing the Chronic Stress Indicator and Allostatic Load 

To better understand some of the critical factors that influence a person’s risk of disease, we propose the CSI. This scale combines sociodemographic factors and biomarkers, to score individuals from low-risk to high-risk (0–10). Factors for this scale include education level, poverty status, alcohol and tobacco use, physical activity, SBP, DBP, TC, HbA1C, and BMI. The ten factors were chosen based on their comprehensive effect on health as indicated by the literature. Using income statistics from the US Department of Health and Human Services, the poverty line ranged from ($17,650–$18,850) over the years 2001–2004. This gives an average poverty line of $18,250 and thus we posit those making less than $20,000 to be considered as living in poverty and are thus classified as high-risk. Additionally, those who obtained less than a high school diploma were deemed high-risk. Individuals who did not participate in physical activities were considered high-risk. We also labeled individuals who consumed alcohol and tobacco as high-risk. The bio/clinical markers deemed necessary were BMI, HBA1C, SBP, DBP, and TC. The high-risk cut offs for these biomarkers were based on being within the top 25 percent of the database’s distribution of the markers. The scores for all ten factors were then summed to obtain the CSI score. Based on the distribution and geometric mean of the CSI score, we considered those with a CSI score of five and above as high-risk for chronic stress. The cut off for high and low AL was determined by the literature [4,23].

Specifically, in terms of allostatic load, it was defined in line with previous studies and our earlier research, through the integration of biomarkers across immune, cardiovascular, and metabolic systems developing an out-of-10 index with an AL value of three considered to be elevated, and thus high-risk, based on the literature. A comparison of the variables included in both CSI and AL indices is shown in Table 1. 

### 2.5. Statistical Analyses

For this study, the statistical evaluations were performed utilizing version 4.1.1 of the R programming language [24] and incorporated the sampling weights from NHANES to account for its sampling design. To begin, an analysis of summary statistics for both AL and CSI identified trends within the dataset was performed. Associations among categorical variables were examined using the chi-square test and Cramer’s V statistic, whereas relationships involving continuous variables were explored through Pearson correlation coefficients. Odds ratios and their 95% confidence intervals were calculated using binary logistic regression models. In all analyses conducted, a *p*-value of less than 0.05 was deemed to indicate statistical significance.

## 3. Results

### 3.1. Overview of Research Participants 

Table 2, Table 3 and Table A1 show the summary statistics for all categorical and continuous variables of AL risk. In choosing variables to assess AL risk, we made sure not to evaluate factors that went into creating the AL as this would bias the results. In Table 2 and Table A1, the rows for continuous variables contain the survey-weighted means as well as the survey-weighted standard errors in parenthesis, whereas the rows for categorical variables show survey-weighted proportions. The *p*-values shown were obtained using the Wilcoxon rank-sum test for complex survey samples for continuous variables and the chi-squared test with the Rao–Scott second-order correction for categorical variables. The AL calculation was based on a sample of 1063 individuals (unweighted), corresponding to a weighted population estimate of 25,098,492. By examining Table 2 and Table A1, we observe a noteworthy correlation between Age and the risk of developing AL. Individuals with a low AL risk tend to be younger on average (average age of 32.28 years) compared to those with a high AL risk (average age of 37.57 years). Furthermore, there is a conspicuous association between AL risk and the duration of OA. Notably, individuals with a low OA duration predominantly fall into the low AL-risk category (59.01%) as opposed to the high AL-risk category (40.99%).

Table A1 is located in Appendix A and presents a detailed analysis of the association between allostatic load (AL) risk and various occupations, among a large population sample (N = 25,098,492). Key findings indicate that occupation is intricately linked with AL.

Table 3 shows the summary statistics for health-related variables grouped by AL risk. We see no significant associations between AL risk and CMV IgG nor Toxoplasma IgG. We also see the majority of those who reported excellent health status were low-risk (70.48%) compared to high-risk (29.52%).

Table 4, Table 5 and Table A2 show the summary statistics for different variables grouped by CSI risk. In these tables, the rows for continuous variables contain the survey-weighted means as well as the survey-weighted standard errors in parenthesis, whereas the rows for categorical variables show survey-weighted proportions. The statistical significance levels (*p*-values) were computed through rigorous methods: for continuous variables, the Wilcoxon rank-sum test was employed, specially adapted for complex survey samples, while categorical variables underwent analysis with the chi-squared test, which incorporated the Rao–Scott second-order correction. In terms of the CSI, the analysis was conducted with data from a sample of 921 individuals (unweighted), which corresponded to a weighted population estimate of 22,862,802.

Table A2 is also located in Appendix A and examines the relationship between the CSI and various occupations in a substantial population (N = 22,862,802). Key findings include significant occupational differences by CSI risk, specifically, occupational activities demonstrate notable relationships with CSI, with significant variations across different occupations. 

Table 5 presents a comprehensive analysis of both socioeconomic/behavioral and health-related variables in relation to Chronic Stress Indicator (CSI) risk. A key observation in the socioeconomic and behavioral aspect is the notable disparity in CSI risk among males, with a significantly higher proportion being categorized as high-risk (66.68%) compared to low-risk (33.32%). This trend is also evident in minority groups, where a higher proportion falls into the high-risk category. Additionally, the data reveals a strong association between occupational activity (OA) and CSI risk. Individuals whose current and longest jobs involve high OA are more likely to be classified as high-risk (67.43% and 76.31%, respectively) compared to those whose jobs involve low OA (32.57% and 23.69%, respectively).

From a health perspective, those deemed high-risk by the CSI have, on average, a higher allostatic load (AL) risk score (3.44) than those as low-risk (1.44). There is also a significant correlation between CSI risk and CMV IgG, as indicated by the *p*-value (0.028), and a similar pattern is observed with Toxoplasma IgG (*p*-value: 0.020). In terms of self-reported health status, the majority of participants who reported poor health were categorized as high-risk by the CSI (97.11%), as opposed to those as low-risk (2.89%). Although not statistically significant, high-risk CSI individuals also reported more days of poor physical (3.94) and mental (4.95) health. This comprehensive view of Table 5 highlights the multifaceted nature of stress as it relates to both socioeconomic/behavioral factors and health outcomes.

### 3.2. Tests of Association

**Figure 1:**
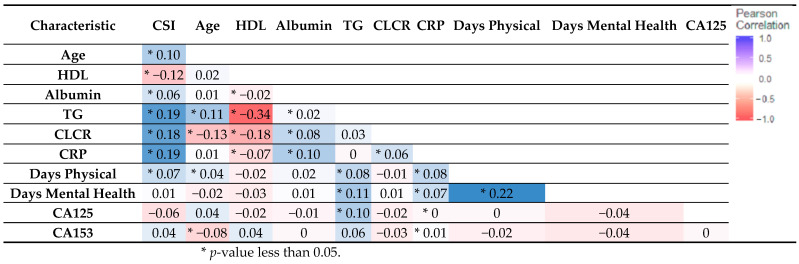
This figure displays Pearson correlation of continuous variables. The correlation coefficients between various health characteristics and CS, including age, HDL, albumin, triglycerides (TG), creatinine clearance rate (CLCR), C-reactive protein (CRP), days of physical activity, and cancer antigens (CA125 and CA153). Notable correlations include a positive association between CSI risk and factors like TG, CRP, and CLCR, while there is a negative correlation with HDL.

To explore additional associations within the dataset, we conducted two types of analyses: Pearson correlation for continuous variables and the Chi-square test of association for categorical variables. Examining the correlation among continuous variables as illustrated in Figure 1, we can discern that HDL and TG exhibited the most robust relationship. Furthermore, we observed notable correlations between each of TG (0.19) and CRP (0.19) with CSI risk, signifying their significant associations. 

**Figure 2:**
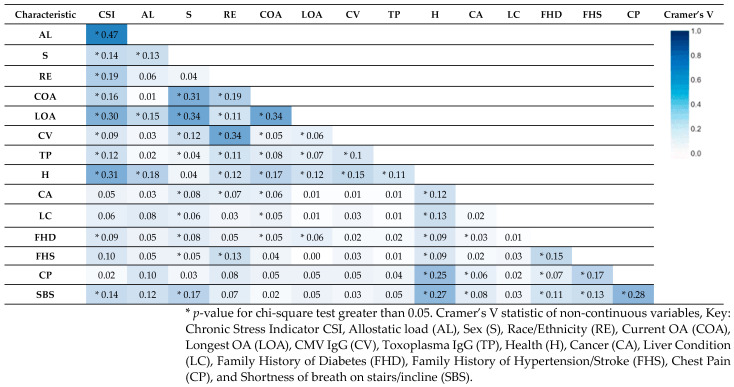
Cramer’s V statistic of categorical variables. This table presents the correlation coefficients between various health characteristics, including CSI, AL, and other critical study variables. Notable correlations include strong associations between the CSI and several variables, particularly AL, COA, LOA, and SBS.

Figure 2 shows Cramer’s V statistic computed for categorical data. We see the strongest association between the CSI and AL of 0.47 which is expected considering they share some biomarkers for their construction. The next two strongest associations with CSI exist between Health abbreviated as ‘H’ (0.31) and LOA (0.30). We also see an association of 0.34 between LOA and COA. 

#### 3.2.1. Variance Inflation Factor

**Figure 3:**
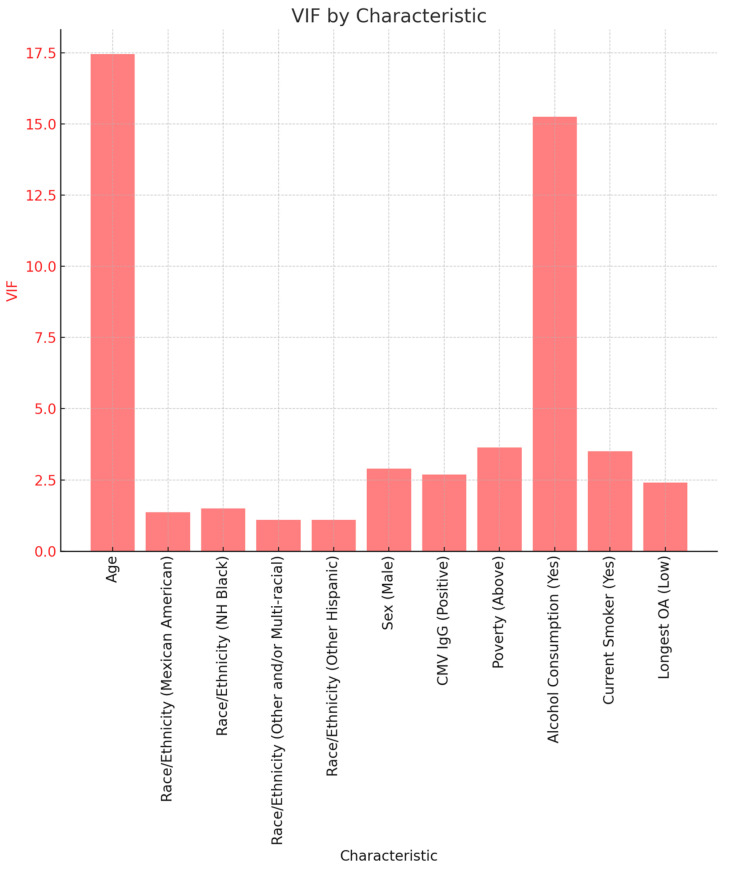
Variance Inflation Factor for critical study variables.

To explore potential multicollinearity, we used the variance inflation factor (VIF). The VIF indicates an increase in variance of coefficients of regression as a result of collinearity. Values greater than 4 or 5 are typically considered indicators of multicollinearity, while a value greater than 10 is considered to be extreme multicollinearity. Figure 3 shows VIF values for some of the variables used in this study. We see that age (17.45) and alcohol consumption (15.25) have the highest VIF. 

#### 3.2.2. Binary Logistic Regression 

Table 6 and Table 7 summarize the results of binary logistic regression models for AL and CSI risk. All predictors for the models were added simultaneously. The analysis of AL risk reveals that an extra year in age corresponds to an average rise of seven percent in the likelihood of experiencing elevated AL. We also notice that compared to females, males have more than double the odds of having high AL. Both of these findings were significant with *p*-values of 0.02 and 0.03, respectively. 

Now we look at the results of the logistic regression model for CSI risk as shown in Table 7. Similar to AL risk, we observe a significant association between CSI risk and age with each additional year in age being associated with an average of a 7% increase in the odds of having a high CSI. Furthermore, we see that smoking is significantly associated with having a high CSI with the odds of smokers developing high CSI risk being more than four times the odds of non-smokers. Additionally, those whose longest occupation contained low OA saw an average reduction of 73% in the odds of having a high CSI. 

Table 8 below summarizes the results of the logistic regression and chi-square test used for comparing the performance between the CSI and AL, in order to ascertain which one predicts critical variables of interest best. A check mark indicates a significantly better predictor while ‘X’ indicates a poor predictor.

#### 3.2.3. Exploratory Factor Analysis (EFA) for AL and CSI

In our study, we applied Exploratory Factor Analysis (EFA) to the sets of variables associated with Allostatic Load (AL) and the Chronic Stress Indicator (CSI) to better understand the underlying structures of these complex constructs (Table 9). The goal was to determine whether it is appropriate to combine these variables into single indices for the AL and CSI, or if a more nuanced approach is needed.

Our analysis revealed that both the AL and CSI are more complex than a single factor can capture. Specifically, for AL, the EFA suggested that up to four distinct factors might be necessary to fully represent the information contained in the variables. This indicates that AL is a multifaceted construct, and a single index might not sufficiently represent its complexity.

For the CSI, while the exact number of factors required for a comprehensive representation was not specified, our results similarly indicate that a single-factor model does not adequately capture all of the nuances of chronic stress as indicated by the variables selected.

In interpreting the EFA Results the chi-square test results, with *p*-values less than 0.05, indicate that adding more factors significantly improves the model fit for both the AL and CSI. This suggests that each construct is multidimensional, necessitating multiple factors to fully capture the variance in the observed variables.

Sum of Squared Loadings (SS Loadings): The SS Loadings for each factor reveal how much variance in the observed variables is explained by that factor. As more factors are added, the cumulative variance explained increases for both the AL and CSI, reinforcing the idea that these constructs are complex and multidimensional.

The cumulative variance explained by the factors increases as more factors are considered, highlighting the importance of recognizing the multidimensional natures of both the AL and CSI for a more accurate analysis.

## 4. Discussion

### 4.1. Allostatic Load as Compared to the Chronic Stress Predictive Model

The goal of this study was to identify a new tool for assessing cumulative stress which we termed the CSI and assess its effectiveness at capturing stress response when compared to a traditional operationalization of chronic stress, the AL. 

Our analysis revealed that age plays a significant role in determining the AL and CSI, for example, a one-unit increase in age corresponded to increased AL. This finding aligns with existing research highlighting the cumulative nature of stress-related physiological changes over a lifespan [25,26]. As individuals age, their bodies may accumulate stress-related damage, potentially leading to a higher AL.

AL predicted biological sex-related stress better than the CSI. This may be because the AL did not consider sociodemographic factors and as such, may not have captured some of the social determinants.

The CSI captured race/ethnicity-related stress better than the AL. This may speak to the fact that race is a social construct and as such, when more social variables are used to assess race/ethnicity-based stress a tool which includes these variables would best capture the manifestation of it. The CSI captured cytomegalovirus and *Toxoplasma gondii* related stress when compared to AL. CMV exposure and disease outcomes are significantly determined by social factors such as household composition, crowding, sexual networks, among other social factors while Toxoplasma gondii is determined by factors such as literacy, unemployment, and water insecurity; thus, it may be correct to say that diseases for which social factors are critical in its exposure and outcomes may require a tool such as the CSI to accurately assess stress-related disease processes [27,28]. 

Disease processes such as shortness of breath and diabetes were best predicted by the CSI when compared with the AL. This may speak to both the biological and social determinants of disease [29]. The CSI, by factoring in both of these factors, is better poised to give a holistic assessment of disease. 

Occupation-related stress was best predicted by the CSI. Given that the CSI includes factors such as income, education, physical activity, alcohol, and tobacco use, its effectiveness in capturing occupation-related stress is likely due to its comprehensive approach. By integrating socioeconomic variables like income and education, along with lifestyle factors such as physical activity and substance use, the CSI offers a nuanced view of how occupational stress interacts with broader life circumstances. This holistic approach of the CSI, encompassing a wide range of influential factors, provides a more complete picture of the stress landscape in various occupational environments [30]. 

Finally, our analysis indicates that the CSI is more effective in capturing occupation-related stress while AL is better at predicting biological sex-related stress. The CSI’s ability to capture occupational-related stress is likely because it includes a comprehensive range of variables such as income, education, physical activity, alcohol, and tobacco use. These factors often correlate closely with different occupational environments and their associated stress levels. In contrast, AL is superior in predicting biological sex-related stress, likely due to its focus on physiological and biological markers. The AL’s lack of emphasis on sociodemographic factors, which could dilute the influence of biological sex, makes it more adept at highlighting sex-based differences in stress responses. This distinction is important for understanding the unique stress profiles associated with different demographic groups and occupational settings.

Overall, the CSI seems to perform better than an operationalization of AL which uses biological-related factors. It must be noted that the direction of social and biological stress-related factors must be considered if one is to consider holistic interventions to mitigate stress-related disease. Namely, do social factors bring forth the biological response or biological factors which result in the biological manifestation of stress contribute to social factors which end up manifesting. To comprehensively answer this question will require further work in the area of epigenomics and other biomedical-related sciences and social sciences to best comprehensively capture the totality of this question. 

In the realm of occupational stress research, the Chronic Stress Predictive Model offers a sophisticated approach to quantifying stress levels by incorporating a range of both environmental and personal factors, aligning well with psychosocial stress theories like the Demand-Control-Support Model [31]. This model postulates that stress results not merely from high demands in the workplace but also from a lack of control over one’s work and insufficient support from colleagues and supervisors. The CSI, by encompassing diverse elements such as income and education, mirrors this model’s multifaceted view of occupational stressors.

Conversely, allostatic load offers a more biologically oriented perspective, focusing on the cumulative wear and tear on the body due to stress. The use of AL as a measure ties closely with concepts like the HPA axis dysregulation model, which explains how prolonged exposure to stress can disrupt normal physiological responses, leading to health issues. This biological focus of AL complements the CSI’s more environmental and social approach, providing a comprehensive understanding of stress that acknowledges both the external pressures individuals face in their occupations and the internal biological responses they trigger.

### 4.2. Broader Implications of Study and Results 

It was critical that we examined chronic stress. Chronic stress has far-reaching consequences on various physiological systems. It can disrupt the immune system, impairing immune responses and increase susceptibility to infections [32]. Inflammatory processes triggered by chronic stress can also contribute to the development of chronic inflammatory conditions, such as rheumatoid arthritis or inflammatory bowel disease. Thus, identifying a better stress response is critical to promoting overall health and wellness. This is made clear by the endocrine system, which is also vulnerable to the impacts of chronic stress, potentially leading to hormonal imbalances and related conditions like insulin resistance and metabolic syndrome [3]. These metabolic disturbances are associated with an increased risk of developing type 2 diabetes and obesity, further underscoring the systemic consequences of chronic stress.

Overall, our comprehensive study needed to include variables such as Socioeconomic status (SES), as it is a key determinant of health outcomes, and its influence on stress levels is significant [33]. A higher SES is associated with better access to resources and opportunities, including quality education, stable employment, and higher income levels, which can reduce stress and promote better health outcomes [6,34,35]. Conversely, a lower SES is linked to increased stress levels due to financial strain, limited educational opportunities, occupational instability, and inadequate access to healthcare. This socioeconomic disadvantage can have profound effects on physical and mental well-being.

The complex interplay between race/ethnicity and SES highlights the disparities in health outcomes among different demographic groups. Racial and ethnic minority populations often face higher levels of poverty, limited access to quality education and healthcare, and increased unemployment rates, all contributing to elevated stress levels and adverse health outcomes [36]. Thus, not accounting for these factors in a measure for stress may fail to capture the complex social determinants of stress. 

Stress can influence various behaviors, including smoking, alcohol consumption, overeating, and reduced physical activity, which can increase the risk of chronic diseases. The relationship between stress and unhealthy behaviors underscores the importance of addressing stress as a contributing factor to poor health choices [37].

Income levels significantly impact stress, with higher income providing a sense of security and access to essential resources, while lower income can lead to constant financial strain, limited access to resources, and heightened stress levels [38]. The stress arising from financial challenges can affect mental and physical well-being.

Higher levels of education are associated with lower stress levels and improved overall well-being [39]. Education empowers individuals with knowledge and skills to navigate life challenges, provides access to better job prospects and higher income, and fosters social connections and support networks, all of which can buffer against stressors.

Occupation plays a pivotal role in determining stress levels [40,41]. High-stress occupations with demanding work environments can lead to elevated stress, burnout, and negative impacts on mental and physical health. In contrast, supportive work environments with job satisfaction and opportunities for growth can mitigate stress levels.

The use of the CSI in various settings such as clinical, occupational, and public health can significantly enhance the assessment and management of chronic stress. In clinical settings, the CSI could be utilized for early identification of individuals at high risk of stress-related conditions, allowing for timely interventions. In occupational environments, this model can inform the design of workplace wellness programs tailored to reduce stressors specific to different job types. In the realm of public health, the CSI can guide policy-making by identifying populations at higher risk due to socioeconomic factors, thereby targeting resources effectively. Practical applications could include developing targeted intervention programs or policies aimed at reducing occupational stress, promoting mental health, and preventing stress-related diseases on a larger scale.

The EFA results challenge the simplicity of summing variables into a single index for both the AL and CSI. They underscore the importance of adopting a multidimensional approach to better reflect the complexity of cumulative stress and its manifestations. Therefore, we recommend the following: Rethinking Index Construction: Future research should consider the multidimensionality revealed by EFA when using the AL and CSI in analyses. This could involve creating separate scores for each identified factor or employing a weighted sum that accounts for the multidimensional structure; Nuanced Measurement: acknowledge the limitations of single-index models and leverage the insights from EFA to develop more nuanced and informative measures of stress-related constructs.

In summary, our findings indicate that the CSI is able to capture a bigger picture in terms of overall factors surrounding the chronic stress of an individual. While we may not definitively say that the CSI is better at capturing chronic stress when compared to AL, we can say that it is worth considering this as well as other alternatives for chronic stress measurement. 

### 4.3. Limitations

This study was a cross-sectional study, and as such, temporality cannot be determined. The findings of this study should be confirmed with longitudinal studies. Future work should also consider analyzing the data using log-binomial methods to avoid potentially introducing a non-collapsibility bias. This study used data from 2001 to 2004 and only included individuals aged 20–49 years old due to the availability of the factors being studied. Future studies should aim to use more recent data and a wider range of participant ages. The factors used to construct the CSI were deemed relevant based on the literature, but future studies may look at other potential factors of cumulative stress. Finally, the adaptation of the CSI in diverse global settings will be critical in future works. This necessitates considering varying socioeconomic and cultural factors unique to each region. The model might require adjustments to reflect different stressors and coping mechanisms prevalent in various cultures. For instance, occupational stressors in a high-income country might differ significantly from those in a developing nation. Cultural perceptions of stress and mental health may also play a crucial role. Therefore, to apply the CSI effectively across different countries, it is essential to incorporate localized data and cultural insights, ensuring the model remains relevant and effective in identifying and managing stress in a global context. Finally, many of the study’s variables may be interrelated in various contexts. Therefore, future research examining these variables should employ more advanced techniques to account for potential multicollinearity. This approach will enhance the model’s applicability across different contexts.

## 5. Conclusions

Our study contributes to the expanding body of knowledge on chronic stress within the U.S. population, emphasizing the role of sociodemographic factors. Our findings indicate that age and sex are significantly correlated with AL, while age, smoking habits, and OA show a notable association with the CSI. The link between smoking and an increased CSI is particularly noteworthy, as smoking is often a coping mechanism for stress. This underscores the importance of promoting healthier stress management strategies, such as reducing smoking and adjusting levels of OA. In defining OA, we categorized jobs into “low” and “high” activity sectors. Low OA roles include positions like executives, administrators, managers, and teachers, while high OA roles encompass jobs such as waiters, cleaners, and laborers in construction and manufacturing. This classification reveals interesting insights into the stress levels experienced by individuals in various occupational fields. Given these insights, our study suggests an urgent need to educate individuals on effective, non-harmful methods for coping with stress. Future research should further explore the array of factors influencing chronic stress. It is crucial for upcoming studies to delve into the interplay between social and behavioral aspects, particularly through the lens of the CSI, to deepen our understanding of how these elements collectively impact stress levels. The nuanced relationship between occupational activity and stress highlights the importance of considering job-related stressors in any comprehensive approach to managing and mitigating chronic stress in the population.

## Figures and Tables

**Table 1:** CSI and AL comparison.

CSI	AL
Systolic Blood Pressure	Systolic Blood Pressure
Diastolic Blood Pressure	Diastolic Blood Pressure
Total cholesterol	Total Cholesterol
Hemoglobin A1c	Hemoglobin A1c
BMI	BMI
Education Level	Triglycerides
Poverty status	High-Density Lipoprotein
Alcohol consumption	Albumin
Tobacco use	Creatinine Clearance
Physical activity	C-Reactive Protein

Note: In this table, the teal color denotes variables that are similar across both indices, while the red color highlights variables that differ.

**Table 2:** Summary statistics of socioeconomic and behavioral variables grouped by AL risk.

		AL Risk		
Characteristic	Overall, N = 25,098,492	Low, N = 13,203,060	High, N = 11,895,432	*p*-Value
Age	34.79 (0.42)	32.28 (0.54)	37.57 (0.49)	<0.001
Sex				<0.001
Female	47.95%	59.28%	40.72%	
Male	52.05%	46.45%	53.55%	
Race/Ethnicity				0.600
NH White	67.96%	52.45%	47.55%	
Mexican American	9.75%	59.34%	40.66%	
NH Black	12.17%	50.50%	49.50%	
Other and/or Multi-racial	5.16%	53.93%	46.07%	
Other Hispanic	4.96%	45.24%	54.76%	
Annual Income				0.072
$0–$4999	4.10%	66.68%	33.32%	
$10,000–$14,999	7.35%	69.41%	30.59%	
$15,000–$19,999	6.76%	50.27%	49.73%	
Under $20,000	0.86%	81.02%	18.98%	
Over $20,000	1.50%	51.31%	48.69%	
$20,000–$24,999	7.06%	54.80%	45.20%	
$25,000–$34,999	11.79%	64.02%	35.98%	
$35,000–$44,999	8.27%	50.22%	49.78%	
$45,000–$54,999	8.43%	55.09%	44.91%	
$5000–$9999	4.10%	47.61%	52.39%	
$55,000–$64,999	7.59%	44.88%	55.12%	
$65,000–$74,999	6.80%	39.55%	60.45%	
$75,000 and over	23.98%	46.83%	53.17%	
Poverty				0.200
At or Below	23.17%	59.92%	40.08%	
Above	75.42%	50.80%	49.20%	
Education				0.200
High school graduate or GED equivalent.	27.59%	54.24%	45.76%	
Grades 9–11 (Includes 12th grade without diploma)	11.47%	44.50%	55.50%	
College graduate or above	20.40%	51.28%	48.72%	
Less than 9th grade	5.09%	42.95%	57.05%	
Some college or AA degree	35.14%	56.11%	43.89%	
Physical Activity				0.004
No	66.75%	46.90%	53.10%	
Unable to Do	0.68%	39.25%	60.75%	
Yes	32.57%	64.57%	35.43%	
Alcohol				0.400
No	24.14%	49.56%	50.44%	
Yes	75.86%	53.58%	46.42%	
Current Smoker				0.500
No	16.94%	45.78%	54.22%	
Yes	29.24%	51.48%	48.52%	

NH—non-Hispanic. This table presents a detailed analysis of the association between allostatic load (AL) risk and various demographic and lifestyle factors, including age, sex, race/ethnicity, income, education, physical activity, alcohol and tobacco use, among a large population sample (N = 25,098,492). Key findings indicate significant associations with age, sex, and physical activity, while other factors like race/ethnicity and income show varied impacts on AL risk.

**Table 3:** Summary Statistics of health-related variables grouped by AL risk.

		AL Risk		
Characteristic	Overall, N = 25,098,492	Low, N = 13,203,060	High, N = 11,895,432	*p*-Value
AL	2.55 (0.09)	1.13 (0.05)	4.13 (0.08)	<0.001
CSI	5.06 (0.12)	4.20 (0.12)	5.93 (0.12)	<0.001
CSI Risk				<0.001
Low	17.96%	78.76%	21.24%	
High	27.04%	30.91%	69.09%	
CMV IgM				0.900
Negative	48.26%	52.11%	47.89%	
Positive	0.73%	56.17%	43.83%	
CMV IgG				0.600
Negative	44.43%	54.01%	45.99%	
Positive	55.42%	51.62%	48.38%	
Toxoplasma IgG Result				0.500
Negative	88.06%	52.19%	47.81%	
Positive	11.94%	55.63%	44.37%	
Toxoplasma IgG	12.39 (1.63)	14.38 (2.62)	10.19 (1.42)	0.700
Cancer Antigen 125	17.38 (0.75)	17.01 (1.09)	17.92 (1.26)	0.700
Cancer Antigen 15.3	2613.28 (404.49)	2312.76 (550.73)	3050.47 (659.93)	0.600
Cancer				0.600
No	96.27%	52.83%	47.17%	
Yes	3.67%	46.07%	53.93%	
Health Status				0.005
Fair	10.57%	43.61%	56.39%	
Excellent	14.09%	70.48%	29.52%	
Good	33.62%	48.27%	51.73%	
Poor	2.30%	26.18%	73.82%	
Very Good	39.42%	53.87%	46.13%	
Liver Condition				0.120
No	96.54%	53.38%	46.62%	
Yes	3.35%	31.12%	68.88%	
Family History of Hypertension/Stroke				0.400
No	57.06%	54.45%	45.55%	
Yes	38.51%	49.73%	50.27%	
Family History of Diabetes				0.200
No	46.25%	55.33%	44.67%	
Yes	51.90%	49.99%	50.01%	
Chest Pain				0.100
No	25.16%	40.77%	59.23%	
Yes	10.58%	30.23%	69.77%	
Shortness of breath on stairs/inclines				0.120
No	25.79%	41.40%	58.60%	
Yes	9.95%	27.92%	72.08%	
Number of days mental health was not good	3.83 (0.30)	3.39 (0.39)	4.32 (0.53)	0.200
Number of days physical health was not good	3.24 (0.30)	2.62 (0.46)	3.93 (0.61)	0.400

AL—Allostatic Load, CSI—Chronic Stress Indicator, CMV—Cytomegalovirus, IgG Immunoglobulin G, and IgM Immunoglobulin M. This table displays the correlation analysis between allostatic load (AL) risk and various health indicators, including stress indices, infection markers, cancer markers, and general health status in a large population sample (N = 25,098,492). It highlights significant differences in AL and chronic stress indices between groups with low and high AL risk, suggesting strong associations with stress and health conditions.

**Table 4:** Summary statistics of socioeconomic and behavioral variables grouped by CSI risk.

		CSI Risk		
Characteristic	Overall, N = 22,862,802	Low, N = 9,088,249	High, N = 13,774,553	*p*-Value
Age	35.36 (0.36)	34.23 (0.66)	36.10 (0.55)	0.056
Sex				<0.001
Female	45.64%	47.42%	52.58%	
Male	54.36%	33.32%	66.68%	
Race/Ethnicity				<0.001
NH White	71.68%	44.76%	55.24%	
Mexican American	8.01%	27.97%	72.03%	
NH Black	9.31%	14.31%	85.69%	
Other and/or multi-racial	4.53%	35.76%	64.24%	
Other Hispanic	6.47%	38.28%	61.72%	
Annual Income				<0.001
$0–$4999	4.50%	25.51%	74.49%	
$10,000–$14,999	7.66%	7.89%	92.11%	
$15,000–$19,999	8.70%	14.62%	85.38%	
Under $20,000	0.99%	4.22%	95.78%	
Over $20,000	1.72%	14.86%	85.14%	
$20,000–$24,999	8.62%	43.40%	56.60%	
$25,000–$34,999	12.05%	50.35%	49.65%	
$35,000–$44,999	9.38%	50.41%	49.59%	
$45,000–$54,999	12.50%	50.99%	49.01%	
$5000–$9999	5.23%	10.03%	89.97%	
$55,000–$64,999	6.02%	55.36%	44.64%	
$65,000–$74,999	4.66%	41.16%	58.84%	
$75,000 and over	17.97%	54.24%	45.76%	

NH—non-Hispanic. This table examines the relationship between CSI and various demographic, income, and health factors in a substantial population (N = 22,862,802). Key findings include significant sex and race/ethnicity disparities in CSI risk, with males and certain ethnic groups showing higher risk. Income levels and occupational activities also demonstrate notable relationships with CSI, with significant variations across different income brackets and occupations.

**Table 5:** Summary Statistics of health-related variables grouped by CSI risk.

		CSI Risk		
Characteristic	Overall, N = 22,862,802	Low, N = 9,088,249	High, N = 13,774,553	*p*-Value
AL	2.64 (0.13)	1.44 (0.14)	3.44 (0.13)	<0.001
AL Risk				<0.001
Low	24.70%	62.86%	37.14%	
High	24.70%	16.95%	83.05%	
CSI	5.04 (0.07)	3.38 (0.03)	6.14 (0.04)	<0.001
HDL	49.67 (0.47)	51.84 (1.01)	48.25 (0.54)	<0.001
Triglycerides	152.76 (11.16)	115.71 (11.40)	177.27 (14.54)	<0.001
Albumin	25.68 (5.12)	10.70 (1.72)	35.62 (7.93)	<0.001
CLCR	144.20 (4.81)	125.11 (6.51)	156.88 (5.66)	<0.001
CRP	0.39 (0.02)	0.22 (0.01)	0.50 (0.03)	<0.001
CMV IgM				0.009
Negative	48.41%	34.62%	65.38%	
Positive	0.81%	82.60%	17.40%	
CMV IgG				0.028
Negative	44.18%	44.63%	55.37%	
Positive	55.66%	36.00%	64.00%	
Toxoplasma IgG Result				0.020
Negative	86.62%	41.97%	58.03%	
Positive	13.38%	25.39%	74.61%	
Toxoplasma IgG	13.74 (2.06)	9.94 (3.14)	16.24 (2.21)	0.055
Cancer Antigen 125	16.19 (0.68)	16.99 (1.24)	15.47 (0.88)	0.200
Cancer Antigen 15.3	3469.03 (518.52)	2890.61 (986.28)	3993.37 (758.96)	0.050
Cancer				0.200
No	96.32%	40.34%	59.66%	
Yes	3.49%	25.77%	74.23%	
Health Status				<0.001
Fair	11.66%	16.02%	83.98%	
Excellent	12.03%	59.96%	40.04%	
Good	35.97%	30.57%	69.43%	
Poor	2.12%	2.89%	97.11%	
Very Good	38.22%	51.32%	48.68%	
Liver Condition				0.200
No	96.43%	40.34%	59.66%	
Yes	3.42%	24.80%	75.20%	
Family History of Hypertension/Stroke				0.077
No	60.47%	44.15%	55.85%	
Yes	34.98%	34.43%	65.57%	
Family History of Diabetes				0.010
No	48.95%	44.71%	55.29%	
Yes	48.60%	35.73%	64.27%	
Chest Pain				0.800
No	25.54%	33.18%	66.82%	
Yes	11.97%	34.82%	65.18%	
Shortness of breath on stairs/inclines				0.025
No	27.37%	37.72%	62.28%	
Yes	10.14%	22.88%	77.12%	
Number of days mental health was not good	4.88 (0.35)	4.77 (0.45)	4.95 (0.37)	0.900
Number of days physical health was not good	3.54 (0.23)	2.92 (0.38)	3.94 (0.39)	0.100

AL—Allostatic Load, CSI—Chronic Stress Indicator, CMV—Cytomegalovirus, IgG Immunoglobulin G, IgM Immunoglobulin M, CRP—C-reactive protein, HDL—High Density Lipoprotein Cholesterol. This table outlines the relationship between CSI risk and various health metrics, including the AL, biochemical markers, infection markers, and general health status in a large sample (N = 22,862,802). It highlights significant differences between AL and CSI indices, lipid profiles (HDL, Triglycerides), and other markers (Albumin, CLCR, CRP) between low and high CSI risk groups. Additionally, it explores the prevalence of certain conditions like cancer, liver disease, and family history of hypertension/stroke and diabetes, alongside subjective health assessments and reports of physical and mental health days.

**Table 6:** Logistic Regression model of AL.

AL Risk		
Characteristic	OR (2.5%, 97.5%)	*p*-Values
Age	1.07 (1.01, 1.12)	0.019
Sex		
Male	2.18 (1.08, 4.38)	0.033
Female	Reference	
Poverty		
Above	0.62 (0.21, 1.82)	0.339
At or Below	Reference	
Alcohol		
Yes	0.37 (0.11, 1.29)	0.106
No	Reference	
Current Smoker		
Yes	0.72 (0.27, 1.92)	0.469
No	Reference	
Longest OA		
Low	0.69 (0.31, 1.56)	0.334
High	Reference	
AIC	312.81	

This table presents Odds Ratios (OR) with 95% Confidence Intervals and *p*-Values for key variables relationship with AL. Notably, age shows a significant association with increased AL risk (OR 1.07, *p* = 0.019). Among gender groups, males have a notably higher AL risk compared to females (OR 2.18, *p* = 0.033). AL—Allostatic Load. OA—Occupational Activity. AIC—Akaike Information Criterion.

**Table 7:** Logistic Regression model of CSI.

CSI Risk		
Characteristic	OR (2.5%, 97.5%)	*p*-Values
Age	1.07 (1.03, 1.11)	0.002
Sex		
Male	1.03 (0.60, 1.75)	0.915
Female	Reference	
Alcohol		
Yes	1.19 (0.45, 3.13)	0.695
No	Reference	
Current Smoker		
Yes	4.70 (2.65, 8.33)	<0.001
No	Reference	
Longest OA		
Low	0.27 (0.11, 0.65)	0.008
High	Reference	
AIC	548.84	

This table presents OR with 95% Confidence Intervals and *p*-Values for Key Variables in Relation to CSI Risk. Age shows a significant association with increased CSI risk (OR 1.07, *p* = 0.002). Sex does not demonstrate a significant difference in risk between males and females. Notably, current smokers have a significantly higher risk of CSI compared to non-smokers (OR 4.70, *p* < 0.001). Low OA is associated with a significantly reduced risk compared to high OA (OR 0.27, *p* = 0.008). Alcohol consumption does not show a significant association with CSI risk. CSI—Chronic Stress Predictive Model. OA—Occupational Activity. AIC—Akaike Information Criterion.

**Table 8:** Comparison of AL and CSI, using logistic regression and chi-square test of association.

	AL	CSI
Age	☑	☑
Sex	☑	X
Race/Ethnicity	X	☑
CMV IgG	X	☑
Toxoplasma IgG	X	☑
Chest Pain	X	X
Shortness of Breath	X	☑
Cancer	X	X
Health	☑	☑
Liver Condition	X	X
FH Diabetes	X	☑
FH Hypertension/Stroke	X	X
Longest Occupation Activity (LOA)	☑	☑
Current Occupational Activity (COA)	X	☑

This table presents logistic regression and chi-square analysis comparing AL and CSI. Significant factors for both AL and CSI include age, health, and LOA. Sex is significant only for AL, while race/ethnicity, CMV IgG, Toxoplasma IgG, and COA (Current Occupational Activity) are unique to CSI. Chest pain, cancer, liver condition, FH of diabetes, and hypertension/stroke show no significant association with either AL or CSI.

**Table 9:** EFA models for CSI and AL.

EFA Models	CSI				AL			
Number of Factors	1 *	2 *	3 *	4	1 *	2 *	3 *	4 *
SS Loadings	1.234	1.012	0.747	0.616	1.271	1.171	1.021	0.790
Cumulative Variance	0.123	0.225	0.299	0.361	0.127	0.244	0.346	0.425

* *p*-value less than 0.05 for chi-square test of number of factors.

## Data Availability

The NHANES dataset is publicly available online, accessible at https://www.cdc.gov/nchs/nhanes/ (accessed on 12 December 2023).

## Appendix A. Occupational Activity

Low occupational activity	High occupational activty
Executive, administrators, and managers	Waiters and waitresses
Management-related occupations	Cleaning and building service occupations
Engineers, architects and scientists	Farm and nursery workers
Teachers	Construction laborers
Secretaries, stenographers, and typists	Construction trades
Information clerks	Laborers, except construction
Records processing occupations	Freight, stock, and material movers, hand
Material recording, scheduling, and distributing clerks	Cooks
Miscellaneous administrative support occupations	Extractive and precision production occupations
Motor vehicle operators	Fabricators, assemblers, inspectors, and samplers
Health diagnosing, assessing and treating occupations	Machine operators, assorted materials
Writers, artists, entertainers, and athletes	Miscellaneous food preparation and service occupations
Other professional specialty occupations	Other helpers, equipment cleaners, hand packagers and laborers
Technicians and related support occupations	Other mechanics and repairers
Supervisors and proprietors, sales occupations	Other transportation and material moving occupations
Farm operators, managers, and supervisors	Protective service occupations
Health service occupations	Related agricultural, forestry, and fishing occupations
Personal service occupations	Textile, apparel, and furnishings machine operators
Private household occupations	Vehicle and mobile equipment mechanics and repairers
Sales representatives, finance, business, & commodities ex. retail	
Sales workers, retail and personal services	
	This table organizes occupations into two main categories based on their activity level. Roles identified as having low occupational activity include those in professional, managerial, administrative, and clerical fields. Examples of such positions are executives, managers, engineers, secretaries, and technicians, typically involving less physical activity. In contrast, occupations classified under high occupational activity are largely made up of manual labor and skilled trades. This group includes construction workers, machine operators, and agricultural workers. Furthermore, occupations in the service industry, such as waiters and cooks, along with those in protective services, are also part of this high-activity category, demanding significant physical effort and continuous vigilance.

**Table A1:** Summary statistics of occupation variables grouped by AL Risk.

		AL Risk		
Characteristic	Overall, N = 25,098,492	Low, N = 13,203,060	High, N = 11,895,432	*p*-Value
Current Occupational Activity (COA)				0.900
High *OA	29.27%	50.34%	49.66%	
Low *OA	48.94%	49.78%	50.22%	
Current Occupation				0.400
Cleaning and building service occupations	1.51%	52.53%	47.47%	
Construction laborers	0.52%	41.28%	58.72%	
Construction trades	6.49%	51.13%	48.87%	
Cooks	0.89%	57.00%	43.00%	
Engineers, architects and scientists	4.26%	49.78%	50.22%	
Executive, administrators, and managers	6.77%	53.19%	46.81%	
Extractive and precision production occupations	0.65%	100.00%	0.00%	
Fabricators, assemblers, inspectors, and samplers	2.15%	48.47%	51.53%	
Farm and nursery workers	0.55%	58.26%	41.74%	
Farm operators, managers, and supervisors	0.32%	0.00%	100.00%	
Freight, stock, and material movers, hand	0.93%	45.38%	54.62%	
Health diagnosing, assessing and treating occupations	1.93%	43.56%	56.44%	
Health service occupations	2.67%	35.23%	64.77%	
Information clerks	0.94%	51.97%	48.03%	
Laborers, except construction	0.21%	100.00%	0.00%	
Machine operators, assorted materials	3.03%	45.46%	54.54%	
Management-related occupations	2.01%	56.72%	43.28%	
Material recording, scheduling, and distributing clerks	1.18%	43.12%	56.88%	
Miscellaneous administrative support occupations	5.45%	63.30%	36.70%	
Miscellaneous food preparation and service occupations	1.65%	45.67%	54.33%	
Motor vehicle operators	1.95%	30.23%	69.77%	
Other helpers, equipment cleaners, hand packagers and laborers	1.23%	44.28%	55.72%	
Other mechanics and repairers	1.19%	33.77%	66.23%	
Other professional specialty occupations	1.44%	50.63%	49.37%	
Other transportation and material moving occupations	1.81%	21.04%	78.96%	
Personal service occupations	1.47%	61.96%	38.04%	
Private household occupations	0.75%	80.97%	19.03%	
Protective service occupations	1.45%	42.91%	57.09%	
Records processing occupations	2.96%	57.98%	42.02%	
Occupations in agriculture, forestry, and fishing-related fields	1.45%	61.87%	38.13%	
Sales representatives, finance, business, & commodities ex. retail	1.87%	28.23%	71.77%	
Sales workers, retail and personal services	3.11%	59.65%	40.35%	
Secretaries, stenographers, and typists	0.88%	56.07%	43.93%	
Supervisors and proprietors, sales occupations	1.47%	45.60%	54.40%	
Teachers	2.09%	30.86%	69.14%	
Technicians and related support occupations	3.52%	50.26%	49.74%	
Textile, apparel, and furnishings machine operators	0.53%	60.70%	39.30%	
Vehicle and mobile equipment mechanics and repairers	1.43%	48.39%	51.61%	
Waiters and waitresses	1.61%	78.96%	21.04%	
Writers, artists, entertainers, and athletes	1.90%	40.07%	59.93%	
Longest Occupational Activity (LOA)				0.005
High *OA	22.24%	43.82%	56.18%	
Low *OA	24.27%	59.01%	40.99%	
Longest Occupation				0.200
Cleaning and building service occupations	1.35%	32.90%	67.10%	
Construction laborers	0.60%	41.43%	58.57%	
Construction trades	1.81%	28.48%	71.52%	
Cooks	0.82%	41.67%	58.33%	
Engineers, architects and scientists	1.05%	52.05%	47.95%	
Executive, administrators, and managers	2.42%	41.83%	58.17%	
Extractive and precision production occupations	2.00%	51.26%	48.74%	
Fabricators, assemblers, inspectors, and samplers	1.23%	49.51%	50.49%	
Farm and nursery workers	0.87%	60.55%	39.45%	
Farm operators, managers, and supervisors	0.56%	0.00%	100.00%	
Freight, stock, and material movers, hand	0.97%	42.47%	57.53%	
Health diagnosing, assessing and treating occupations	1.05%	60.14%	39.86%	
Health service occupations	1.58%	57.54%	42.46%	
Information clerks	0.73%	88.87%	11.13%	
Laborers, except construction	0.73%	31.42%	68.58%	
Machine operators, assorted materials	1.53%	35.26%	64.74%	
Management-related occupations	0.80%	72.24%	27.76%	
Material recording, scheduling, and distributing clerks	0.23%	26.71%	73.29%	
Military occupations	0.22%	0.00%	100.00%	
Miscellaneous administrative support occupations	2.78%	48.99%	51.01%	
Miscellaneous food preparation and service occupations	1.50%	67.38%	32.62%	
Motor vehicle operators	1.27%	40.95%	59.05%	
Other helpers, equipment cleaners, hand packagers and laborers	0.36%	38.39%	61.61%	
Other mechanics and repairers	1.07%	36.39%	63.61%	
Other professional specialty occupations	0.82%	59.27%	40.73%	
Other transportation and material moving occupations	0.99%	24.01%	75.99%	
Personal service occupations	0.90%	93.33%	6.67%	
Private household occupations	0.29%	53.69%	46.31%	
Protective service occupations	0.68%	68.17%	31.83%	
Records processing occupations	0.78%	100.00%	0.00%	
Related agricultural, forestry, and fishing occupations	1.23%	42.48%	57.52%	
Sales representatives, finance, business, & commodities ex. retail	0.24%	53.36%	46.64%	
Sales workers, retail and personal services	3.69%	66.31%	33.69%	
Secretaries, stenographers, and typists	1.02%	89.52%	10.48%	
Supervisors and proprietors, sales occupations	0.68%	61.42%	38.58%	
Teachers	0.53%	88.23%	11.77%	
Technicians and related support occupations	1.67%	53.44%	46.56%	
Textile, apparel, and furnishings machine operators	0.83%	62.30%	37.70%	
Vehicle and mobile equipment mechanics and repairers	1.11%	65.87%	34.13%	
Waiters and waitresses	2.34%	35.97%	64.03%	
Writers, artists, entertainers, and athletes	1.17%	44.37%	55.63%	

* OA—Occupational Activity.

**Table A2:** Summary statistics of Occupational variables grouped by CSI Risk.

		CSI Risk		
Characteristic	Overall, N = 22,862,802	Low, N = 9,088,249	High, N = 13,774,553	*p*-Value
Current Occupational Activity (OA)				0.005
High OA	32.08%	32.57%	67.43%	
Low OA	43.73%	49.04%	50.96%	
Current Occupation				0.049
Cleaning and building service occupations	1.44%	58.71%	41.29%	
Construction laborers	0.65%	18.98%	81.02%	
Construction trades	7.43%	28.44%	71.56%	
Cooks	0.58%	16.10%	83.90%	
Engineers, architects and scientists	3.08%	58.19%	41.81%	
Executive, administrators, and managers	6.15%	66.52%	33.48%	
Extractive and precision production occupations	2.48%	44.42%	55.58%	
Fabricators, assemblers, inspectors, and samplers	1.39%	15.77%	84.23%	
Farm and nursery workers	0.51%	34.87%	65.13%	
Farm operators, managers, and supervisors	0.23%	100.00%	0.00%	
Freight, stock, and material movers, hand	0.74%	0.00%	100.00%	
Health diagnosing, assessing and treating occupations	1.50%	69.96%	30.04%	
Health service occupations	2.53%	21.83%	78.17%	
Information clerks	0.81%	51.20%	48.80%	
Laborers, except construction	0.18%	37.33%	62.67%	
Machine operators, assorted materials	2.47%	51.24%	48.76%	
Management-related occupations	0.86%	74.98%	25.02%	
Material recording, scheduling, and distributing clerks	1.92%	25.21%	74.79%	
Miscellaneous administrative support occupations	6.35%	54.59%	45.41%	
Miscellaneous food preparation and service occupations	1.70%	43.84%	56.16%	
Motor vehicle operators	2.59%	29.88%	70.12%	
Other helpers, equipment cleaners, hand packagers and laborers	0.89%	8.01%	91.99%	
Other mechanics and repairers	2.03%	39.15%	60.85%	
Other professional specialty occupations	1.00%	71.07%	28.93%	
Other transportation and material moving occupations	1.55%	13.41%	86.59%	
Personal service occupations	0.94%	57.99%	42.01%	
Private household occupations	0.17%	100.00%	0.00%	
Protective service occupations	1.27%	23.38%	76.62%	
Records processing occupations	2.49%	40.25%	59.75%	
Related agricultural, forestry, and fishing occupations	1.72%	38.96%	61.04%	
Sales representatives, finance, business, & commodities ex. retail	2.02%	37.44%	62.56%	
Sales workers, retail and personal services	3.88%	33.62%	66.38%	
Secretaries, stenographers, and typists	0.72%	46.55%	53.45%	
Supervisors and proprietors, sales occupations	1.59%	12.50%	87.50%	
Teachers	1.30%	57.63%	42.37%	
Technicians and related support occupations	2.36%	39.92%	60.08%	
Textile, apparel, and furnishings machine operators	0.69%	0.00%	100.00%	
Vehicle and mobile equipment mechanics and repairers	1.96%	35.40%	64.60%	
Waiters and waitresses	2.40%	40.01%	59.99%	
Writers, artists, entertainers, and athletes	1.22%	100.00%	0.00%	
Longest OA				0.003
High OA	27.46%	23.69%	76.31%	
Low OA	24.68%	53.16%	46.84%	
Longest Occupation				0.023
Cleaning and building service occupations	1.48%	33.39%	66.61%	
Construction laborers	1.17%	0.00%	100.00%	
Construction trades	2.93%	19.95%	80.05%	
Cooks	0.54%	48.25%	51.75%	
Engineers, architects and scientists	1.09%	47.19%	52.81%	
Executive, administrators, and managers	2.43%	53.66%	46.34%	
Extractive and precision production occupations	2.77%	7.66%	92.34%	
Fabricators, assemblers, inspectors, and samplers	1.86%	34.56%	65.44%	
Farm and nursery workers	0.57%	24.72%	75.28%	
Farm operators, managers, and supervisors	0.16%	0.00%	100.00%	
Freight, stock, and material movers, hand	1.57%	26.33%	73.67%	
Health diagnosing, assessing and treating occupations	0.49%	46.52%	53.48%	
Health service occupations	1.50%	40.69%	59.31%	
Information clerks	0.77%	70.90%	29.10%	
Laborers, except construction	1.19%	6.02%	93.98%	
Machine operators, assorted materials	1.55%	41.71%	58.29%	
Management-related occupations	1.49%	54.84%	45.16%	
Material recording, scheduling, and distributing clerks	0.49%	0.00%	100.00%	
Military occupations	0.23%	0.00%	100.00%	
Miscellaneous administrative support occupations	3.49%	52.96%	47.04%	
Miscellaneous food preparation and service occupations	1.80%	20.76%	79.24%	
Motor vehicle operators	1.38%	9.67%	90.33%	
Other helpers, equipment cleaners, hand packagers and laborers	0.41%	10.13%	89.87%	
Other mechanics and repairers	1.09%	31.46%	68.54%	
Other professional specialty occupations	0.51%	69.41%	30.59%	
Other transportation and material moving occupations	1.52%	16.27%	83.73%	
Personal service occupations	1.13%	22.74%	77.26%	
Private household occupations	0.15%	0.00%	100.00%	
Protective service occupations	0.60%	8.44%	91.56%	
Records processing occupations	1.02%	79.77%	20.23%	
Related agricultural, forestry, and fishing occupations	1.29%	28.28%	71.72%	
Sales representatives, finance, business, & commodities ex. retail	0.69%	83.88%	16.12%	
Sales workers, retail and personal services	2.27%	34.28%	65.72%	
Secretaries, stenographers, and typists	0.98%	51.23%	48.77%	
Supervisors and proprietors, sales occupations	1.04%	63.27%	36.73%	
Teachers	0.50%	100.00%	0.00%	
Technicians and related support occupations	1.85%	85.42%	14.58%	
Textile, apparel, and furnishings machine operators	0.93%	7.35%	92.65%	
Vehicle and mobile equipment mechanics and repairers	1.77%	32.87%	67.13%	
Waiters and waitresses	2.19%	44.24%	55.76%	
Writers, artists, entertainers, and athletes	1.26%	87.74%	12.26%	

OA—Occupational Activity.
